# Road map for, and technical challenges of, carbon-nanotube integrated circuit technology

**DOI:** 10.1093/nsr/nwad261

**Published:** 2023-10-10

**Authors:** Jia Si, Panpan Zhang, Zhiyong Zhang

**Affiliations:** Key Laboratory for the Physics and Chemistry of Nanodevices and Center for Carbon-based Electronics, School of Electronics, Peking University, China; State Key Laboratory of Information Photonics and Optical Communications, Beijing University of Posts and Telecommunications, China; Key Laboratory for the Physics and Chemistry of Nanodevices and Center for Carbon-based Electronics, School of Electronics, Peking University, China; State Key Laboratory of Information Photonics and Optical Communications, Beijing University of Posts and Telecommunications, China

## Abstract

A new targeted observational algorithm was developed to optimize prediction targets across various regions and variables. This approach was utilized to design an optimal ENSO monitoring array in the TPOS 2020 project.

The past decade has witnessed an explosion of research on carbon-nanotube (CNT) electronics due to their outstanding properties, including an ultrathin body, a high carrier mobility and a high saturation velocity. CNT field-effect transistors (CNT FETs) with a simple planar gate structure have been shown to maintain extraordinary normalized performance under an ultrascaled gate length of 5 nm, which provides a promising strategy for going beyond the limits of silicon complementary metal–oxide–semiconductor (CMOS) integrated circuit (IC) technology [1]. However, the fabrication of high-performance CNT-based ICs and the realization of the full potential of CNTs are still challenging. From this perspective, a road map for CNT technology is proposed in the context of transistor architecture, material engineering and process innovations to fully unleash the intrinsic potential of CNTs.

The performance advantages of CNTs over silicon technology are evaluated at the block level (101-stage ring oscillator) for several technology nodes (N90/N28/N5), taking the energy-delay product (EDP) as the primary figure of merit (FOM). The well-established Stanford virtual source and Berkeley short-channel IGFET model (BSIM) models for CNT and silicon FETs, respectively, are used to implement the simulations. Figure 1 illustrates that the EDP of CNT technology outperforms that of the silicon counterpart by 44.5$\times $, 55.4$\times $ and 30.3$\times $ at the N90, N28 and N5 nodes, respectively. Challenges and expected innovations to accelerate CNT technology node development to attain the targeted EDP are set forth below.

**Figure 1.**
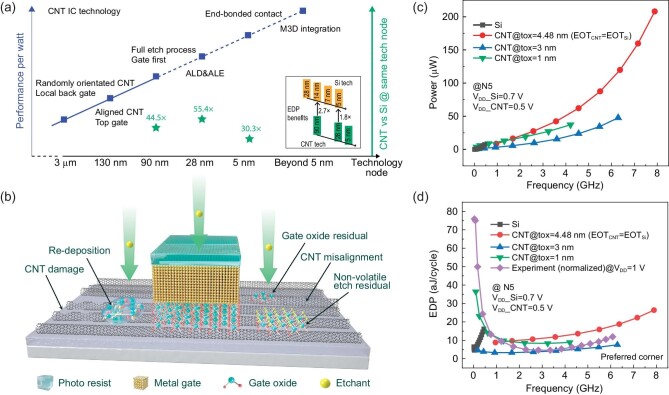
(a) Technology and performance road map for CNT ICs. (b) Illustration of non-ideal situations during the CNT IC dry etching process, including CNT damage and misalignment, gate dielectric residue and etching residue. (c) Comparison of the projected power consumption of CNT ICs with that of Si ICs at the N5 technology node. (d) Comparison of the projected performance of CNT ICs with that of Si ICs at the N5 technology node.

A CNT FET requires pure semiconducting (>99.9999%) and dense parallel CNTs (100–200 tubes/μm) with a specific diameter in the channel to supply a sufficient driving current [2]. Randomly oriented CNT networks (or films) were adopted to fabricate micrometer-scale ICs in the early stage for the sake of wafer-scale uniformity, whereas the performance and scalability were hindered by the junctions between different segments of CNTs. When thorough purification and controllable positioning of CNTs into aligned arrays became feasible, top-gated CNT FETs with a channel length of 120 nm (130 nm CNT technology) achieved an on-state current of 1.3 mA/μm and a record transconductance of 0.9 mS/μm. The CNT ring oscillators at this node exhibited a higher performance (speed) than the commercial Si CMOS ICs under a comparable gate length for the first time [3]. For a CNT FET in which well-aligned CNTs are assembled in the channel, a wide distribution of diameters will lead to a reduced bandgap and asynchronous turn-on for different nanotubes, thereby degrading the subthreshold swing (SS) characteristic and causing serious leakage of transistors. To alleviate this issue, CNTs with a specific diameter, or a single chirality, are highly favored from the material perspective. CNTs of different diameters can be used for high performance or low power consumption. In addition, device mechanism and structure innovations can be employed to suppress the leakage problem of CNT FETs caused by their small bandgap [4,5].

We consider N90 as the optimal entry point for special commercial applications such as radiation-hardened ICs and sensors, given that CNT N90 can provide larger driving current and EDP than silicon N28 (experimental results) [6], which currently holds over 75% of the market share in the semiconductor industry. However, fabricated FETs are now facing severe SS degradation and poor reliability issues. The SS is not easily suppressed to below 100 mV/decade through a normal gate insulator due to the charge trapping/detrapping events occurring between carriers and trap states at the interface (D_it_ > 10^12^ eV^−1 ^cm^−2^) or oxide layer. There are two major ways to fabricate P-type and N-type CNT FETs. One is through doping-free technology by applying metals with different work functions as the source and drain contacts, i.e. Pd for P-type FET and Sc for N-type FET [3,6,7]. The other is through channel doping by using HfO2, Al2O3, AlN, etc. as the capping layer [8]. Doping-free technology achieves better performance; however, source/drain contact metals (Pd for p-FET or Sc for n-FET) cannot be sustained in high-temperature atomic layer deposition (ALD) (>300°C), which was initially designed to remove defects within high-k dielectrics. Moreover, contact oxidation occurs when oxygen or water vapor enters the interface between the Sc contact and the aligned CNT film in the ALD process [7]. All the above issues can be alleviated by developing a gate-first structure and a full etch process, implying that the fabrication of CNT ICs must be transferred from the laboratory to industry. However, the most challenging problem for all low-dimensional semiconductor-based FETs is to etch the gate stacks such that the etching stops exactly at the monolayer channel surface without destroying it while ensuring that all gate dielectric residues are removed as shown in Fig. 1b. There should be a co-optimization of the high-k metal gate stack and etching recipe considering the target threshold voltage as well as etching residues. A proper wet cleaning process is needed to remove the residue of dry etching byproducts to form good ohmic contacts. In addition, etching recipes for special metals used in CNT ICs (Pd, Sc, Y and so on) need to be developed from scratch. Developing a customized cleaning process for CNT IC technology, including CNT materials and fabrication processes, is essential. Substrate pretreatment, gas annealing, solution cleaning and so on can be used to alleviate the impurities adsorbed on CNT materials.

Extreme scaling of CNT FETs down to N28 and N5 requires meticulous surface control, demanding the development of distinctive process alongside innovative equipment. This includes developing an atomic-layer-etching (ALE) technique to form good CNT/high-k and CNT/contact surfaces and developing mild but efficient cleaning techniques. End-bonded contact technology is also expected to be incorporated at the 5 nm technology node or earlier [9]. Unlike the silicon CMOS, which has to adopt fin field-effect transistor (FinFET) or gate-all-around (GAA) architectures to maintain superior gate control for scaled nodes at the cost of process complexity, a plain planar architecture is sufficient for N5 CNT FETs at lower supply voltages. The power consumption and EDP of CNT ICs at N5 show great benefits with respect to the same-node Si ICs, as shown in Fig. 1c and d, while CNT N28 can still surpass silicon technology by 1.8x at N5.

The scalability and energy efficiency of CNT ICs could be further improved by using a monolithic three-dimensional (M3D) architecture beyond the 5 nm technology node. The deposition of CNT films is free of substrates, and the process temperature of CNT FETs is lower than 400°C. These advantages enable the ‘real’ 3D integration of CNT electronics with various technologies into one chip through ultradense interlayer vias (ILVs, scaling toward sub-100 nm). Recently, a breakthrough has been achieved in M3D CNT ICs utilizing two-layer FETs, which have a comparable performance and a reduced-by-half area with respect to the best planar CNT circuits [10]. Another promising application is the near-memory computing system, which integrates CNT logic and state-of-the-art memories into one chip to save latency and energy during data movements [11]. Furthermore, multifunctional systems that include e.g. sensing, storage, computing and communication capabilities utilizing CNT M3D technology will very likely be implemented soon. Despite the numerous advantages of CNT ICs, their lifespan and reliability remain areas that require further study and improvement.

In summary, the development of CNT ICs is still in its infancy, and collaborative efforts from both industry and academia are urgently needed in the future.
